# Fremanezumab in the prevention of high-frequency episodic and chronic migraine: a 12-week, multicenter, real-life, cohort study (the FRIEND study)

**DOI:** 10.1186/s10194-022-01396-x

**Published:** 2022-04-09

**Authors:** Piero Barbanti, Gabriella Egeo, Cinzia Aurilia, Florindo d’Onofrio, Maria Albanese, Ilaria Cetta, Paola Di Fiore, Maurizio Zucco, Massimo FilippiBonassi, Francesco Bono, Claudia Altamura, Stefania Proietti, Stefano Bonassi, Fabrizio Vernieri, for the FRIEND-Study Group

**Affiliations:** 1 Headache and Pain Unit, IRCCS San Raffaele Pisana, Rome, Italy; 2 San Raffaele University, Rome, Italy; 3 Neurology Unit, San Giuseppe Moscati Hospital, Avellino, Italy; 4 Regional Referral Headache CenterNeurology Unit, University Hospital Tor Vergata, Rome, Italy; 5 Department of Systems Medicine, University of Rome Tor Vergata, Rome, Italy; 6 Headache Unit, Department of Neurology, Scientific Institute San Raffaele Hospital, Vita-Salute University, Via Olgettina, 48, Milan, Italy; 7 Headache Center, ASST Santi Paolo Carlo, Milan, Italy; 8 Headache Center, Neurlogy Unit, San Camillo-Forlanini Hospital, Rome, Italy; 9 Neurology Unit, IRCCS San Raffaele Scientific Institute, Vita-Salute San Raffaele University, Milan, Italy; 10 Center for Headache and Intracranial Pressure Disorders, Neurology Unit, A.O.U. Mater Domini, Catanzaro, Italy; 11 Headache and Neurosonology Unit, Policlinico Universitario Campus Bio-Medico, Rome, Italy; 12 Clinical and Molecular Epidemiology, IRCCS San Raffaele, Roma, Italy; 13 Department of Human Sciences and Quality of Life Promotion, San Raffaele University, Rome, Italy; 14 Headache and Neurosonology Unit, Campus Bio-Medico University Hospital, Rome, Italy

**Keywords:** Fremanezumab, Migraine treatment, CGRP monoclonal antibody, Real-world, Predictor

## Abstract

**Background:**

Fremanezumab has demonstrated to be effective, safe, and tolerated in the prevention of episodic or chronic migraine (CM) in randomized, placebo-controlled trials (RCTs). Real-life studies are needed to explore drug effects in unselected patients in routine circumstances and to provide higher generalizability results. This study explores the effectiveness, safety, and tolerability of fremanezumab in a real-life population of individuals affected by high-frequency episodic (HFEM: 8–14 days/month) or CM.

**Methods:**

This is a 12-week multicenter, prospective, cohort, real-life study. We considered all consecutive patients affected by HFEM or CM visited at 9 Italian headache centers from 28/07/2020 to 11/11/2020. Eligible patients were given subcutaneous fremanezumab at the doses of 225 mg monthly or 675 mg quarterly, according to their preference. Primary study endpoints were the change in monthly migraine days (MMDs) in HFEM and monthly headache days (MHDs) in CM patients at weeks 9–12 compared to baseline. Secondary endpoints encompassed variation in monthly analgesic intake (MAI), Numerical Rating Scale (NRS), HIT-6 and MIDAS scores, and ≥ 50%, ≥ 75% and 100% responder rates at the same time intervals.

**Results:**

Sixty-seventh number migraine patients had received ≥ 1 subcutaneous fremanezumab dose and were considered for safety analysis, while 53 patients completed 12 weeks of treatment and were included also in the effectiveness analysis. Fremanezumab was effective in both HFEM and CM, inducing at week 12 a significant reduction in MMDs (-4.6, *p* < 0.05), MHDs (-9.4, *p* < 0.001), MAI (-5.7, *p* < 0.05; -11.1, *p *< 0.001), NRS (-3.1, *p* < 0.001; -2.5, *p* < 0.001), and MIDAS scores (-58.3, *p* < 0.05; -43.7; *p* < 0.001). HIT-6 was significantly reduced only in HFEM patients (-18.1, *p* < 0.001). Remission from CM to episodic migraine and from MO to no-MO occurred in 75% and 67.7% of the patients. The ≥ 50%, ≥ 75% and 100% responder rates at week 12 were 76.5%, 29.4% and 9.9% in HFEM and 58.3%, 25% and 0% in CM. Younger age emerged as a positive response predictor (OR = 0.91; 95% CI 0.85–0.98, *p* = 0.013). Treatment-emergent adverse events were uncommon (5.7%) and mild. No patient discontinued fremanezumab for any reason.

**Conclusions:**

Fremanezumab seems more effective in real-life than in RCTs. Younger age emerges as a potential response predictor.

**Supplementary Information:**

The online version contains supplementary material available at 10.1186/s10194-022-01396-x.

## Introduction

Migraine is a complex neurologic disorder characterized by recurrent disabling headache episodes associated with autonomic symptoms [1]. The conventional oral preventive migraine therapies are non-selective, non-specific, poorly tolerated, and burdened by a high discontinuation rate [2–4]. Monoclonal antibodies (mAbs) targeting the calcitonin gene-related peptide (CGRP)—the first specific migraine prophylactic agents—are changing the scene of migraine prevention, coupling promising efficacy to an excellent tolerability profile [5]

Fremanezumab is a humanized mAbs targeting both the α and β CGRP isoforms, indicated for the prevention of episodic or chronic migraine in adults. Its peculiarity is the flexible dose regimen which allows to personalize the treatment choosing between the dose of 225 mg on monthly basis or 675 mg quarterly [6]. Fremanezumab has been extensively investigated in randomized, placebo-controlled trials (RCTs) in patients affected by episodic migraine (EM) (HALO-EM study), chronic migraine (CM) (HALO-CM study) and EM or CM with 2 to 4 prior therapeutic failures (FOCUS study), documenting a significant superiority over placebo in reducing migraine frequency, analgesic use and disability, and a good efficacy/tolerability profile also in long-term treatment trials [7–10].

While pharmacological RCTs test drugs under ideal conditions, real-life studies explore their effectiveness, safety, and tolerability in unselected patients in routine circumstances, detecting rare or late-onset adverse events, assessing adherence and patterns of use, providing higher generalizability results, and testing new hypotheses [11]. In addition to the growing real-life evidence on erenumab [12, 13] and galcanezumab [14, 15], preliminary results from non-peer reviewed publications on retrospective, real-world studies are available for fremanezumab, documenting a reduction ranging from 68.7% to 77% in monthly migraine days (MMDs) and from 65.9% to 74.8% in monthly headache days (MHDs) in EM or CM, lower acute medication use and emergency department, and outpatient physician costs [16].

The present paper is aimed at evaluating the effectiveness, safety, and tolerability of fremanezumab in a prospective real-life, multicenter Italian study in patients affected by high-frequency episodic (HFEM: ≥ 8 MMDs) or CM.

## Methods

This is a 12-week multicenter, prospective, cohort, real-life study ongoing at 9 headache centers distributed across 4 Italian regions (Lombardy, Latium, Campania, and Calabria) from July 28^th^, 2020, with the latest data analysis performed on November 11^th^, 2020. We considered all consecutive patients affected by HFEM or CM—according to the criteria of the International Classification of Headache Disorders, 3rd edition [17]—with indication to fremanezumab preventive treatment according to the reimbursement rules of the Italian Medicine Agency (AIFA) [18]. None of them was previously treated with any antiCGRP mAbs.

After signing the informed consent, all patients underwent a careful physical and neurological examination and were interviewed using a shared semi-structured questionnaire by specifically trained, board-certified neurologists who gathered information on socio-demographic characteristic, migraine features, past and current migraine treatments, comorbidities, and concomitant medications [12].

Patients were given subcutaneous fremanezumab at the doses of 225 mg monthly or 675 mg quarterly, according to their preference. During the 28-day run-in baseline period and the entire study duration, patients were asked to fill-out a paper–pencil diary recording MMDs for HFEM, MHDs for CM, monthly analgesic intake, and rating pain intensity of the monthly most painful attack (0–10, Numerical Rating Scale, NRS). A migraine day was defined as a calendar day characterized by > 4 consecutive hours of a migraine with or without aura or a headache of any duration successfully treated with migraine-specific acute medications (triptans). Pain disability was measured monthly using the Headache Impact Test (HIT-6) and quarterly with the Migraine Disability Assessment Scale (MIDAS).

The primary study endpoints were the change in MMDs for HFEM and MHDs for CM at weeks 9–12 compared to baseline. Secondary endpoints encompassed variation in monthly analgesic intake, NRS, HIT-6 and MIDAS scores and ≥ 50%, ≥ 75% and 100% response rates at the same time intervals. All adverse events (AEs) were evaluated.

The study, not preregistered, was approved by the IRCCS San Raffaele Roma Institutional Review Board (RP 19/26) as coordinating center and mutually recognized by the other local Institutional Review Boards.

## Statistical methods

Categorical variables in demographic and clinical data were reported as percentages, and group differences were assessed using the χ^2^ test or the Fisher exact test (2-tailed). Continuous data was summarized as mean and standard deviation (SD) for descriptive purposes. The comparison of post treatment values with baseline was done with the paired *t*-test or non-parametric Wilcoxon test for paired data, while the comparison of groups of patients with episodic or chronic migraine was done with the independent Student’s *t*-test or by the Mann Whitney U test if the distribution of the data was non normal. The Shapiro–Wilk test was applied to test the departure from normality of data distribution. A multivariate logistic regression models was fitted to identify factors associated with the response. Potential confounders and variables which were statistically significant in the univariate analysis were included in the models. Risks were expressed as odds ratio (OR) along with its 95% confidence interval (95% CI). To consider the possibility that a single center might have driven statistical analysis, a sensitivity analysis was performed, re-evaluating the effects of treatment on selected endpoints (MMDs, monthly analgesic intake, VAS, HIT-6 and MIDAS scores) after removing one-by-one each clinical center. This approach clearly showed that none of the center was an influential data point, and this variable was no longer considered in the statistical analysis. Results were considered statistically significant when *p* < 0.05. SPSS (IBM SPSS Statistics for Windows, version 27.0) and GraphPad Prism (GraphPad Software, Inc; v8.00.) statistical software was used for statistical analysis.

## Results

Sixty-seven migraine patients (HFEM, *n* = 21; CM, *n* = 46; F/M 53/14; mean age 48.4 years) at November 11^th^ 2020 had received at least one subcutaneous fremanezumab dose (225 mg monthly, *n*

**Fig. 1:**
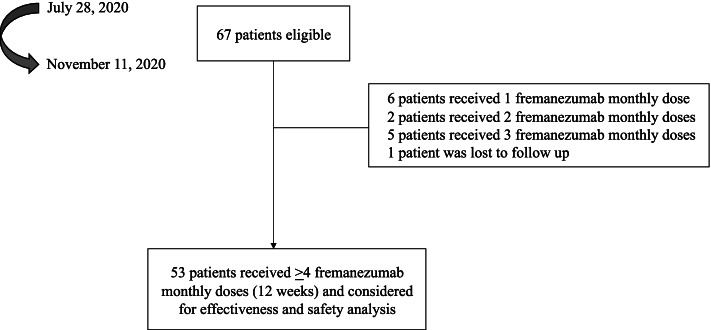
Patients’ disposition

 = 58; 625 mg quarterly, *n* = 9) and were considered for safety analysis, while 53 patients completed 12 weeks of treatment and were included in the effectiveness analysis (Fig. 1). Table 1 summarizes their demographic and clinical features. Most patients were females (41/53, 77.3%), affected by CM (36/53, 67.9%), with concomitant medication overuse (MO: 31/36, 86.1%) and showed on average 4.5 prior preventative failures. The only variable which showed significant difference between the two groups was the monthly analgesic intake, which was nearly double in CM as compared to HFEM (22.4 ± 18.5 vs 11.9 ± 7.1; *p* < 0.029). Only 9 patients (17%) were treated with a quarterly fremanezumab dosing regimen.

**Table 1:** Demographic and clinical features of patients with high-frequency episodic migraine (HFEM) or chronic migraine (CM)

	**All Patients**	**HFEM**	**CM**	***P*-value**
**Patients**	53	17	36	
**Age,** *yrs*, mean ± SD	47.7 ± 11.5	47.5 ± 11.6	47.9 ± 11.6	ns
**Females,** n (%)	41 (77.3)	12 (70.5)	29 (80.5)	ns
**BMI,** mean ± SD	24.0 ± 3.6	23.6 ± 2.4	24.2 ± 4.1	ns
**Age at CM onset,** mean ± SD		-	34.0 ± 13.1	-
**Disease duration*,**** yrs*, mean ± SD	29.6 ± 13.9	29.1 ± 13.0	29.8 ± 14.4	ns
**MMDs/MHDs at baseline**, mean ± SD	17.0 ± 6.2	10.5 ± 1.8	20.0 ± 5.2	-
**NRS score,** mean ± SD	8.6 ± 1.1	8.5 ± 1.2	8.6 ± 1.1	ns
**Pain location,** n (%)				
*Unilateral*	21 (44.7)	8 (50)	13 (41.9)	ns
*Unilateral, bilateral*	23 (48.9)	8 (50)	15 (48.4)	
*Bilateral*	3 (6.4)	0	3 (9.7)	
**Pain quality,** n (%)				
*Pulsating*	28 (57.1)	8 (53.3)	20 (58.8)	ns
*Pressing/tightening*	12 (24.5)	3 (20.0)	9 (26.5)	
*Other*	9 (18.4)	4 (26.7)	5 (14.7)	
**Unilateral cranial autonomic symptoms,** n (%)	33 (62.2)	12 (70.5)	21 (58.3)	ns
**Allodynia,** n (%)	29 (59.2)	10 (66.7)	19 (55.8)	ns
**Dopaminergic symptoms,** n (%)	30 (61.2)	10 (66.7)	20 (58.8)	ns
**Monthly analgesic intake,** mean ± SD	19.0 ± 16.5	11.9 ± 7.1	22.4 ± 18.5	**0.029**
**MO,** n (%)		-	31 (86.1)	-
**Duration of MO,** *yrs*, mean ± SD		-	24.8 ± 35.0	-
**Triptan responders,** n (%)	34 (64.1)	12 (70.6)	22 (61.1)	ns
**Pts using concomitant prophylaxis,** n (%)	29 (54.7)	7 (41.1)	22 (61.1)	ns
*Tricyclics*	10 (34.5)	2 (28.5)	8 (36.4)	
*Anticonvulsants*	7 (24.1)	2 (28.5)	5 (22.7)	
*Calcium-antagonists*	1 (3.4)	0	1 (4.5)	
*Serotoninergic antagonists*	5 (17.2)	0	5 (22.7)	
*Beta-blockers*	9 (31.0)	2 (28.5)	7 (31.8)	
*BoNT/A*	2 (6.9)	0	2 (9.1)	
*Other*	6 (20.7)	2 (28.5)	4 (18.2)	
**Prior treatment failures,** mean ± SD	4.5 ± 2.3	3.9 ± 1.5	4.7 ± 2.5	ns
*1–2*	4 (8.0)	3 (17.7)	1 (3.0)	
*3–4*	30 (60.0)	10 (58.8)	20 (60.6)	
> *4*	16 (32.0)	4 (23.5)	12 (36.4)	
**BoNT/A responders**^*a*^, n (%)	2 (11.1)	2 (33.3)	0	0.098
**Pts with ≥ 1 comorbidity,** n (%)	34 (64.2)	14 (82.4)	20 (55.5)	0.072
**Pts with psychiatric comorbidities,** n (%)	10 (19.2)	3 (18.8)	7 (19.4)	ns
**HIT-6 score,** mean ± SD	65.2 ± 17.2	68.2 ± 3.4	63.7 ± 20.8	ns
**MIDAS score,** mean ± SD	89.4 ± 48.9	78.9 ± 50.5	94.5 ± 48.1	ns
**Fremanezumab dosing regimen,** n (%)				
*Monthly*	44 (83.0)	13 (76.5)	31 (86.1)	ns
*Quarterly*	9 (17.0)	4 (23.5)	5 (13.9)	

*HFEM* High frequency episodic migraine, *CM* Chronic migraine, *BMI* Body mass index, *MMDs* Monthly migraine days, *MHDs* Monthly headache days, *NRS *Numerical rating scale, *MO*   Medication overuse, *BoNT/A *Onabotulinum toxin A, *HIT-6* Headache Impact Test-6, *MIDAS* Migraine disability assessment test

^a^Proportion calculated on the 18 subjects who were treated with *BoNT/A*

**Fig. 2:**
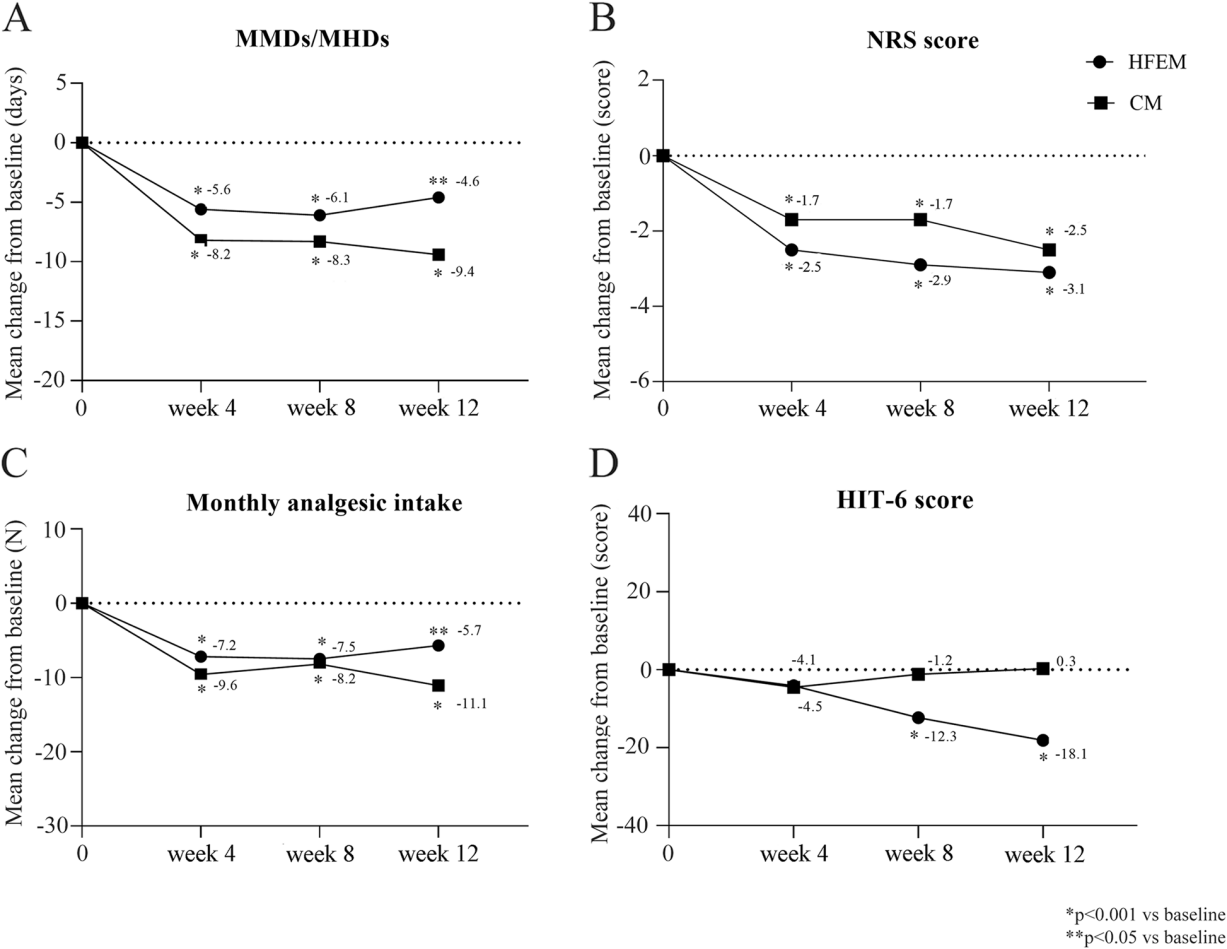
Mean change in **A** monthly migraine days/monthly headache days (MMDs/MHDs), **B** Numerical Rating Scale (NRS), **C** monthly analgesic intake, and **D** Headache Impact Test-6 (HIT-6) score from baseline to Week 12. CM, chronic migraine; HFEM, high-frequency episodic migraine

Fremanezumab was effective in both HFEM and CM patients (Fig. 2, supplementary table 1). At weeks 4, 8 and 12, fremanezumab induced in HFEM patients a significant reduction in MMDs (-5.6 ± 2.9, *p* < 0.001; -6.1 ± 3.6, *p* < 0.001; -4.6 ± 6.5, *p* < 0.05), monthly analgesic use (-7.2 ± 6.7, *p* < 0.001; -7.5 ± 5.8, *p* < 0.001; -5.7 ± 6.6, *p* < 0.05), NRS score (-2.5 ± 2.5, *p* < 0.05; -2.9 ± 1.9, *p* < 0.001; -3.1 ± 2.5, *p* < 0.001) and HIT-6 score (-4.1 ± 10.2, ns; -12.3 ± 5.5, *p* < 0.001; -18.1 ± 13.2, *p* < 0.001). At the same time intervals, fremanezumab significantly (*p* < 0.001 for all) reduced MHDs (-8.2 ± 6.1; -8.3 ± 6.8; -9.4 ± 6.9), monthly analgesic use (-9.6 ± 13.1; -8.2 ± 9.2; -11.1 ± 14.2) and NRS score (-1.7 ± 1.8; -1.7 ± 1.7; -2.5 ± 2.7) in patients with CM. HIT-6 scores did not significantly vary in CM (-4.5 ± 21.3; -1.2 ± 20.1; 0.3 ± 23.3). MIDAS score at weeks 9–12 was significantly reduced in HFEM (-58.3 ± 57.7; *p* < 0.05) and CM patients (-43.7 ± 63.4; *p* < 0.001) (Fig. 2, supplementary table 1).

Sixty-one-point-one percent (22/36) of CM patients remitted to episodic migraine at week 4, 72.2% (26/36) at week 8, and 75% (27/36) at week 12, while 61.2% (19/31) of the patients with MO remitted to no-MO at week 4, 64.5% (20/31) at week 8, and 67.7% (21/31) at week 12. Remission from CM to episodic migraine was sustained across week 8 in 100% (22/22), and week 12 in 95.5% (21/22) of the patients, while remission from MO to no-MO was sustained in 94.7% (18/19), and 94.4% (17/18) of the cases, respectively (Table 2).

**Table 2:** Patients remitting from chronic migraine (CM) to episodic migraine (EM) and from medication overuse (MO) to no medication overuse (no-MO) across weeks 4, 8 and 12 following fremanezumab treatment

	**Week 4**	**Week 8**	**Week 12**
**CM remission to EM**	22/36 (61.1%)	26/36 (72.2%)	27/36 (75%)
* Sustained remission across weeks 4, 8, 12*	-	22/22 (100%)	21/22 (95.5%)
**MO remission to no-MO**	19/31 (61.2%)	20/31 (64.5%)	21/31 (67.7%)
* Sustained remission across weeks 4, 8, 12*	-	18/19 (94.7%)	17/19 (94.4%)

**Fig. 3:**
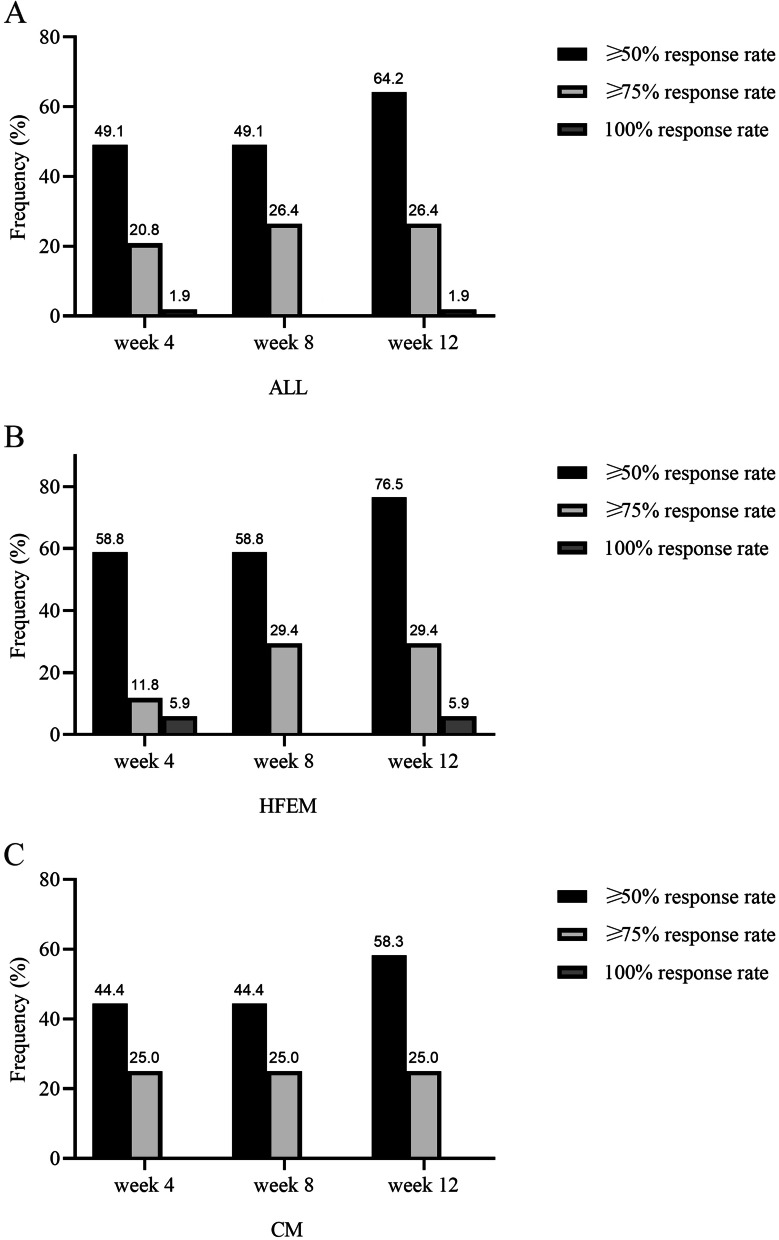
Response rates at week 4, week 8, and week 12 in the global patients’ population (ALL), patients with high-frequency episodic migraine (HFEM), and chronic migraine (CM)

The ≥ 50%, ≥ 75% and 100% responder rates at week 12 were 76.5%, 29.4% and 9.9% in HFEM and 58.3%, 25% and 0% in CM patients (Fig. 3). Safety and tolerability data were provided by 60 out of the 67 patients treated with ≥ 1 fremanezumab dose because at the time of data analysis 7 patients had not yet performed the first follow-up visit, scheduled 4 weeks after the first dose administration. Treatment-emergent adverse events, rated as mild and transient, were reported by 1 patient at week 4 (1.7%), 2 at week 8 (3.4%) and 3 (5.7%) at week 12 (Table 3). The most common was injection site erythema. No patient discontinued fremanezumab treatment for any reason.

**Table 3:** Treatment-emergent adverse event (TEAEs) occurring at weeks 4, 8 and 12

	**Week 4**	**Week 8**	**Week 12**
Patients n	60	58	53
**Patients with ≥ 1 TEAE**	1 (1.7%)	2 (3.4%)	3 (5.7%)
•*Injection site erythema*	1 (1.7%)	1 (1.7%)	1 (1.9%)
•*Dizziness*	-	1 (1.7%)	-
•*Abdominal pain*	-	-	1 (1.9%)
•*Neck pain and somnolence*	-	-	1 (1.0%)
**Discontinuation due to TEAEs**	-	-	-
**Discontinuation due to ineffectiveness**	-	-	-

The univariate analysis of determinant of ≥ 50% response documented that responders were significantly younger (44.3 ± 11.3 vs 53.8 ± 9.4 years; *p* = 0.03), had lower monthly analgesic intake (15.3 ± 15.0 vs 25.7 ± 17.3; *p* = 0.026), shorter medication overuse duration (13.8 ± 12.1 vs 40.4 ± 49.5 months; *p* < 0.041), and more frequent use of the monthly dosing regimen (75% vs 11%; *p* = 0.001) (Table 4).

**Table 4:** Univariate analysis of independent determinant of ≥ 50% response

	** < 50% response**	** > 50% response**	***P*-value**
**Patients,** n (%)	19 (35.8)	34 (64.2)	-
**Age,** yrs, mean ± SD	53.8 ± 9.4	44.3 ± 11.3	**0.03**
**Females**, n (%)	14 (34.1)	27 (65.9)	ns
**BMI,** mean ± SD	24.4 ± 3.9	23.7 ± 3.5	ns
**Age at CM onset,** mean ± SD	37.2 ± 15.4	31.9 ± 11.2	ns
**Disease duration,** mean ± SD	31.5 ± 15.0	28.5 ± 13.3	ns
**MMDs/MHDs at baseline,** mean ± SD	18.9 ± 6.3	15.9 ± 6.0	ns
**NRS score,** mean ± SD	8.6 ± 1.3	8.5 ± 1.0	ns
**Pain location, n (%)**			ns
* Unilateral*	8 (44.4)	13 (44.8)	
* Unilateral, bilateral*	8 (44.4)	15 (51.7)	
* Bilateral*	2 (11.2)	1 (3.5)	
**Pain quality, n (%)**			ns
* Pulsating*	9 (56.3)	19(57.6)	
* Pressing/tightening*	6 (37.5)	6 (18.2)	
* Other*	1 (6.2)	8 (24.2)	
**UAs,** n (%)	9 (56.3)	24 (72.7)	ns
**Allodynia**, n (%)	10 (62.5)	19 (57.6)	ns
**Dopaminergic symptoms,** n (%)	10 (62.5)	20 (60.6)	ns
**MAI at baseline,** mean ± SD	25.7 ± 17.3	15.3 ± 15.0	**0.026**
**MO**, n (%)	13 (86.7)	18 (85.7)	ns
**Duration of MO**, months; mean ± SD	40.4 ± 49.5	13.8 ± 12.1	**0.041**
**Triptan responders,** n (%)	12 (63.2)	22 (64.7)	ns
**Pts using concomitant prophylaxis**, n (%)	11 (57.9)	18 (52.9)	ns
* Tricyclics*	4 (36.4)	6 (33.3)	
* Anticonvulsants*	3 (27.3)	3 (16.7)	
* Calcium-antagonists*	0	1 (5.6)	
* Serotoninergic antagonists*	1 (9.1)	4 (22.2)	
* Beta-blockers*	3 (27.3)	6 (33.3)	
* BoNT/A*	0	2 (11.1)	
* Other*	2 (18.2)	4 (22.2)	
**Prior treatment failures,** mean ± SD	5.1 ± 3.1	4.1 ± 1.5	ns
1–2	2 (10.5)	2 (6.5)	
3–4	9 (47.4)	21 (63.6)	
> 4	8 (42.1)	8 (24.2)	
**Response to BoNT/A,** n (%)	4 (50.0)	7 (70.0)	ns
**Pts with ≥ 1 comorbidity,** n (%)	11 (32.4)	23 (67.6)	ns
**Pts with psychiatric comorbidities,** n (%)	3 (15.8)	7(21.2)	ns
**HIT-6 score at baseline,** mean ± SD	69.1 ± 4.1	62.7 ± 21.5	ns
**MIDAS score at baseline,** mean ± SD	93.7 ± 42.0	86.9 ± 52.9	ns
**Fremanezumab dosing regimen,** n (%)			
* Monthly*	11 (57.9)	33 (97.1)	**0.001**
* Quarterly*	8 (42.1)	1 (2.9)	

*CM* Chronic migraine, *BMI* Body mass index, *MMDs* Monthly migraine days, *MHDs* Monthly headache days, *NRS* Numerical rating scale, *UAs* Unilateral cranial autonomic symptoms, *MAI*   Monthly analgesic intake, *MO*  Medication overuse, *BoNT/A*   Onabotulinum toxin A, *HIT-6* Headache Impact Test-6, *MIDAS*  Migraine disability assessment test

When stratifying patients according to migraine frequency, younger age and shorter medication overuse emerged as positive predictors only in CM (*p* < 0.001 and 0.041, respectively), whereas lower monthly analgesic intake was found only in HFEM patients (*p* = 0.046). Monthly dosing regimen was associated to higher probability of fremanezumab responsiveness in both HFCM (*p* = 0.022) and CM patients (*p* = 0.008), although the small number of responders among those treated quarterly makes these statistics highly unstable. Lastly, CM patients with lower HIT-6 score were more likely to be fremanezumab responders (*p* = 0.038). To consider the role of confounders, a multivariate logistic regression analysis model was fitted to data. The only variable which survived the backward process was age (OR = 0.91; 95% CI 0.85–0.98, *p* = 0.013), confirming the results of univariate analysis, which showed a better response rate in younger patients. The effect of fremanezumab dosing regimen could not be properly estimated because of the small number of subjects treated quarterly.

## Discussion

Real-life studies are needed to translate the evidence concerning the efficacy of antiCGRP mAbs into effectiveness, to confirm their safety and tolerability, and to identify response predictors in a multifaceted clinical setting. This approach is particularly valuable to study patients with various comorbidities and inadequate response to diverse classes of prior preventive treatments.

The present prospective, multicenter, real-life Italian study documents that fremanezumab is effective, safe, and well tolerated in the prevention of subjects affected by difficult-to-treat HFEM or CM with multiple therapeutic failures. These results demonstrate an early and progressive improvement in migraine frequency, analgesic use, pain intensity, and MIDAS score in these patients. Of note, the treatment induced the remission from CM to episodic migraine and from MO to no-MO in over two-thirds of the patients, the improvement being persistent over 12 weeks in almost all cases. Adverse events were extremely rare, and no patient discontinued the treatment for any reason.

In published RCTs focusing on patients with < 2 prior therapeutic failures, fremanezumab demonstrated to be significantly superior to placebo in reducing MMDs (HALO-EM: -3.7/-3.4 vs -2.2; *p* < 0.001) and MHDs (HALO-CM: -4.6/-4.3; *p* < 0.001) as well as in ≥ 50% responder rate (HALO-EM: 47.7%/44.4% vs 27.9%, *p* < 0.0001; HALO-CM: 40.8%/37.6% vs 18.1%; *p* < 0.0001), revealing also a good safety and tolerability profile [7, 8]. Interestingly, fremanezumab proved an even better efficacy/tolerability ratio in patients who had not responded to 2–4 preventive medication clusters (FOCUS study), documenting a higher therapeutic gain over placebo in terms of MMDs/MHDs change (-4.1/-3.7 vs -0.6; *p* < 0.0001) and ≥ 50% responder rate (34% vs 9%, *p* < 0.0001) when compared to the HALO-EM and HALO-CM trials, coupled to a lower incidence of adverse events (45%-55% vs 66.2%-66.3% and 70%-71%, respectively) [9].

Patients considered in the FRIEND study were harder to treat than those included in the FOCUS trial because almost all of them had failed ≥ 3 preventive treatments (93% vs 53%), had higher migraine frequency (18.9 days/month vs 14.3 days/month), were more frequently affected by CM (67.9% vs 60.1%) and MO (86.1 vs 52%), and showed higher disability (MIDAS score 89.4 vs 61.8). Further, among episodic migraine patients, we considered only HFEM, i.e., those with ≥ 8 MMDs.

A direct comparison of real-life data with the results of RCTs is not scientifically accurate, especially when the number of patients studied is heterogeneous. In addition, differences in placebo and nocebo effects may affect anti-CGRP mAbs treatment success in different study settings. This notwithstanding, it’s worth mentioning that at weeks 9–12 the reduction in MMDs/MHDs and the proportion of ≥ 50% responders in our patients were greater than those reported in the FOCUS study (-7.9 vs -3.7/-4.1; 64.2% vs 34%, respectively), suggesting that the effectiveness of fremanezumab could be better than its efficacy. We reported similar findings also for erenumab and galcanezumab in the EARLY and GARLIT real-world studies [12–15], where we documented a nearly twofold increase in the proportion of ≥ 50% responders at 12 weeks (~ 60% and ~ 70%, respectively) compared to the LIBERTY and CONQUER studies [20, 21]. Why antiCGRP mAbs seem to work better in migraine patients with more complex clinical picture (as the real-life ones) compared to those enrolled in the RCTs, is matter of speculation. This could depend—at least in part—on their higher baseline MMDs/MHDs. As a matter of fact, migraine frequency positively correlates with CGRP plasma levels, which are higher in CM than in episodic migraine patients and healthy controls [22]. Notably, even among real-life patients, we documented a better antiCGRP mAbs responsiveness in those with higher baseline migraine frequency (OR: 1.12; 95%CI: 1.05–1.20, *p* < 0.001) [13]. Another possible reason is the high proportion of patients with allodynia in real-life (59.2% in our study). Allodynia is seen as a CGRP-related symptom and prevails in patients with long-lasting attacks, long disease history, severe pain intensity and disability, MO, and psychiatric comorbidities, features typically characterizing the real-world setting [23–25]. For the above reasons, it can be argued that real-life patients could be characterized by a greater CGRP pathogenetic involvement, thus being more sensitive to CGRP-targeting treatments.

In the FRIEND study, fremanezumab significantly improved MIDAS score in all the patients, but reduced HIT-6 only in those with HFEM. We have no reliable explanation for this controversial finding, probably biased by the small number of patients studied. MIDAS and HIT-6 are complimentary in measuring migraine-related disability but also show some substantial differences. MIDAS is basically a function of migraine frequency and relies on concrete variables (i.e., days of work or school missed), whilst HIT-6 depends more on headache severity and explores patients’ impressions on how migraine is affecting them [26].

Consequently, we cannot exclude that the above discrepancy is also somehow related to the different benefits produced by fremanezumab on migraine frequency and intensity in our patients’ groups. Indeed, at week 12, the reduction in MMDs/MHDs was comparable in HFEM (-47%,) and CM (-43.8%), whereas the decrease in intensity (NRS) was more evident in episodic (-36.5%) than in chronic migraine patients (-29%).

Age came up as a potential negative response predictor in our study (OR = 0.91; 95% CI 0.85–0.98, *p* < 0.013). The odd of being fremanezumab responder would decrease by 9% for each year of age, suggesting that its effectiveness could be higher in younger patients. This assumption, however, must be considered with caution, because the population studied is small and mostly represented by CM patients. Similarly, the apparent clinical advantage of the monthly dosing regimen over the quarterly one cannot be established because most patients (83%) had preferred a monthly 225 mg fremanezumab dose.

The results of the present study should be interpreted with caution due to some limitations. The patient sample is small, due to limited pre-reimbursement access to fremanezumab in our Country (in Italy the reimbursement is now limited to adult patients with > 8 MMDs over the last 3 months, MIDAS score > 11, and documented failure, contraindications, or low tolerability to > 3 pharmacological classes of migraine preventive medications among beta-blocker, anticonvulsants and tryciclics, or onabotulinum toxinA for CM). Some comparisons between responders and non-responders were based on very small samples, such in the case of treatment regimen. The limited size of these analyses recommends the use of p-values to achieve information about the credibility of the result rather than for hypothesis testing. Lastly, patients reported migraine data using paper–pencil diaries, a less reliable tool compared to modern electronic diaries. Strengths are the multicenter prospective design, the involvement of 9 headache centers representative of northern, central, and southern Italy and a careful clinical characterization of the patients through a shared detailed semi-structured questionnaire.

In conclusion, our multicenter, prospective study indicates that fremanezumab is effective and extremely tolerated also in real-life subjects affected by HFEM or CM with multiple therapeutic failures. Our results deserve confirmation in larger real-life studies.

## Supplementary Information

**Additional file 1: Table S1.** Change in monthly migraine days (MMDs), monthly headache days (MHDs), monthly analgesic intake, Numerical Rating Scale (NRS) score, Headache Impact Test-6 (HIT-6) score, and Migraine Disability Assessment Scale (MIDAS) score from baseline to week 12. **Table S2.** Univariate analysis of independent determinant of ≥50% response in patients with high-frequency episodic migraine (HFEM) or chronic migraine (CM).

## Data Availability

Anonymized data will be shared by request from any qualified investigator.

## Abbreviations

CM: Chronic migraine
RCTs: Placebo-controlled trials
HFEM: High-frequency episodic
MMDs: Monthly migraine days
MAI: Monthly analgesic intake
NRS: Numerical Rating Scale
HIT-6: Headache Impact Test
MIDAS: Migraine Disability Assessment Scale
mAbs: Monoclonal antibodies
CGRP: Calcitonin gene-related peptide
EM: Episodic migraine
AEs: Adverse events
SD: Standard deviation
