# Correction: Fremanezumab in the prevention of high-frequency episodic and chronic migraine: a 12-week, multicenter, real-life, cohort study (the FRIEND study)

**DOI:** 10.1186/s10194-022-01434-8

**Published:** 2022-06-07

**Authors:** Piero Barbanti, Gabriella Egeo, Cinzia Aurilia, Florindo d’Onofrio, Maria Albanese, Ilaria Cetta, Paola Di Fiore, Maurizio Zucco, Massimo Filippi, Francesco Bono, Claudia Altamura, Stefania Proietti, Stefano Bonassi, Fabrizio Vernieri, for the FRIEND-Study Group

**Affiliations:** 1 Headache and Pain Unit, IRCCS San Rafaele Pisana, Rome, Italy; 2 San Rafaele University, Rome, Italy; 3 Neurology Unit, San Giuseppe Moscati Hospital, Avellino, Italy; 4 Regional Referral Headache CenterNeurology Unit, University Hospital Tor Vergata, Rome, Italy; 5 Department of Systems Medicine, University of Rome Tor Vergata, Rome, Italy; 6 Headache Unit, Department of Neurology, Scientifc Institute San Rafaele Hospital, Vita-Salute University, Via Olgettina, 48, Milan, Italy; 7 Headache Center, ASST Santi Paolo Carlo, Milan, Italy; 8 Headache Center, Neurlogy Unit, San Camillo-Forlanini Hospital, Rome, Italy; 9 Neurology Unit, IRCCS San Rafaele Scientifc Institute, Vita-Salute San Raffaele University, Milan, Italy; 10 Center for Headache and Intracranial Pressure Disorders, Neurology Unit, A.O.U. Mater Domini, Catanzaro, Italy; 11 Headache and Neurosonology Unit, Policlinico Universitario Campus Bio-Medico, Rome, Italy; 12 Clinical and Molecular Epidemiology, IRCCS San Rafaele, Roma, Italy; 13 Department of Human Sciences and Quality of Life Promotion, San Rafaele University, Rome, Italy; 14 Headache and Neurosonology Unit, Campus BioMedico University Hospital, Rome, Italy

**Correction: J Headache Pain 23, 46 (2022)**

**https://doi.org/10.1186/s10194-022-01396-x**

Following the publication of the original article [1], we were notified of a mistake in the abstract: the 100% response rate in HFEM at week 12 is 5.9% (as correctly reported in figure 3) and NOT 9.9% as reported in the abstract and page 5.

The original article has been corrected.
