# Supplementary material for: Fremanezumab in the prevention of high-frequency episodic and chronic migraine: a 12-week, multicenter, real-life, cohort study (the FRIEND study)
Source: J Headache Pain. 2022 Apr 9;23(1):46. doi: 10.1186/s10194-022-01396-x (PMC8994302; doi:10.1186/s10194-022-01396-x)
Supplement: Supplementary file 1 — Additional file 1: Table S1. Change in monthly migraine days (MMDs), monthly headache days (MHDs), monthly analgesic intake, Numerical Rating Scale (NRS) score, Headache Impact Test-6 (HIT-6) score, and Migraine Disability Assessment Scale (MIDAS) score from baseline to week 12. Table S2. Univariate analysis of independent determinant of ≥50% response in patients with high-frequency episodic migraine (HFEM) or chronic migraine (CM). [file 10194_2022_1396_MOESM1_ESM.docx]

**Table S1.** Change in monthly migraine days (MMDs), monthly headache days (MHDs), monthly analgesic intake, Numerical Rating Scale (NRS) score, Headache Impact Test-6 (HIT-6) score, and Migraine Disability Assessment Scale (MIDAS) score from baseline to week 12.

*p<0.05 **p<0.001

|  | **HFEM** | | | | **CM** | | | |
| --- | --- | --- | --- | --- | --- | --- | --- | --- |
|  | **Baseline** | **Week 4** | **Week 8** | **Week 12** | **Baseline** | **Week 4** | **Week 8** | **Week 12** |
| **MMDs** *(Mean ± SD)*  *Change from baseline* | 10.5±1.8  - | 4.9±2.4  -5.6±2.9^**^ | 4.5±2.8  -6.1±3.6^**^ | 5.9±6.8  -4.6±6.5^*^ | - | - | - | - |
| **MHDs** *(Mean ± SD)*  *Change from baseline* | - | - | - | - | 20.0±5.2  - | 11.7±7.  -8.2±6.1^**^ | 11.7±8.6  -8.3±6.8^**^ | 10.6±7.8  -9.4±6.9^**^ |
| **Monthly analgesics intake** *(Mean ± SD)*  *Change from baseline* | 11.9±7.1  - | 4.6±2.7  -7.2±6.7^**^ | 4.3±3.7  -7.5±5.8^**^ | 6.2±7.  -5.7±6.6^*^ | 22.4±18.5  - | 13.4±15.7  -9.6±13.1^**^ | 14.1±16.6  -8.2±9.2^**^ | 11.3±10.8  -11.1±14.2^**^ |
| **NRS score** *(Mean ± SD)*  *Change from baseline* | 8.5±1.2  - | 6.1±2.1  -2.5±2.5^*^ | 5.6±1.5  -2.9±1.9^**^ | 5.4±2.1  -3.1±2.5^**^ | 8.6±1.1  - | 6.8±1.8  -1.7±1.8^**^ | 6.9±1.6  -1.7±1.7^**^ | 6.1±2.3  -2.5±2.7^**^ |
| **HIT-6** *(Mean ± SD)*  *Change from baseline* | 68.2±3.4 | 64.1±3.4  -4.1±10.2 | 56.2±5.5  -12.3±5.5^**^ | 50.6±14.5  -18.1±13.2^**^ | 63.7±20.8 | 58.6±7.1  -4.5±21.3 | 61.8±9.6  -1.2±20.1 | 60.9±8.2  0.3±23.3 |
| **MIDAS** *(Mean ± SD)*  *Change from baseline* | 78.9±50.5 | - | - | 21.0±11.6  -58.3±57.7^*^ | 94.5±48 | - | - | 52.1±69.9  -43.7±63.4^**^ |

**Table S2**. Univariate analysis of independent determinant of ≥50% response in patients with high-frequency episodic migraine (HFEM) or chronic migraine (CM)

|  | **HFEM** | | | **CM** | | |
| --- | --- | --- | --- | --- | --- | --- |
|  | **<50% response** | **>50% response** | ***p*-value** | **<50% response** | **>50% response** | ***p*-value** |
| **Patients,** n (%) | 4 (23.5) | 13 (76.5) | - | 15 (41.7) | 21 (58.3) | **-** |
| **Age,** yrs, mean±SD | 49.0±11.0 | 47.1±12.2 | ns | 55.1±8.9 | 42.7±10.6 | **0.001** |
| **Females,** n (%) | 2 (50.0) | 10 (76.9) | ns | 12 (80.0) | 17 (80.9) | ns |
| **BMI,** mean±SD | 24.2±3.4 | 23.3±2.5 | ns | 24.4±4.4 | 24.0±4.0 | ns |
| **Age at CM onset,** mean±SD | - | - | - | 37.2±15.4 | 31.9±11.2 | ns |
| **Disease duration,** mean±SD | 20.3±6.2 | 31.8±13.5 | ns | 34.5±15.3 | 26.5±13.1 | ns |
| **MMDs at baseline,** mean±SD | 10.7±2.5 | 10.5±1.7 | ns | 21.1±5.1 | 19.3±5.2 | ns |
| **NRS score,** mean±SD | 9.3±0.9 | 8.3±1.3 | ns | 8.5±1.4 | 8.7±0.9 | ns |
| **Pain location**, n (%)  *Unilateral*  *Unilateral, bilateral*  *Bilateral* | 2 (50.0)  2 (50.0)  - | 6 (50.0)  6 (50.0)  - | ns | 6 (42.9)  6 (42.9)  2 (14.2) | 7 (41.1)  9 (52.9)  1 (6.0) | ns |
| **Pain quality, n (%)**  *Pulsating*  *Pressing/tightening*  *Other* | 1 (33.3)  1 (33.3)  1 (33.3) | 7(58.3)  2(16.7)  3 (25.0) | ns | 8 (61.5)  5 (38.5)  - | 12 (57.1)  4 (19.0)  5 (23.8) | ns |
| **UAs,** n (%) | 2 (66.7) | 10 (83.3) | ns | 7 (53.8) | 14 (66.7) | ns |
| **Allodynia,**n (%) | 1 (33.3) | 9 (75.0) | ns | 9 (69.2) | 10 (47.6) | ns |
| **Dopaminergic symptoms,** n (%) | 3 (100) | 7 (58.3) | ns | 7 (53.8) | 13 (61.9) | ns |
| **MAI at baseline,** mean±SD | 18.0±11.7 | 10.0±4.2 | **0.046** | 27.7±18.3 | 18.5±18.2 | ns |
| **MO**, n (%) | - | - | - | 13 (86.7) | 18 (85.7) | ns |
| **Duration of MO,** yrs, mean±SD | - | - | - | 40.4±49.5 | 13.8±12.1 | **0.041** |
| **Triptan responders**, n (%) | 3 (75.0) | 9 (69.2) | ns | 9 (60.0) | 13 (61.9) | ns |
| **Concomitant prophylaxis,** pts n (%)  *Tricyclics*  *Anticonvulsants*  *Calcium-antagonists*  *Serotoninergic antagonists*  *Beta-blockers*  *BoNT/A*  *Other* | 3 (75.0)  3(100)  1 (33.3)  1 (33.3)  0  0  1(33.3)  0  0 | 4 (30.8)  4 (100)  1 (25.0)  1 (25.0)  0  0  1(25.0)  0  2(50.0) | ns | 8(53.3)  8 (100)  3 (37.5)  2 (25.0)  0  1 (12.5)  2 (25.0)  0  2(50.0) | 14 (66.7)  14 (100)  5 (35.7)  3 (21.4)  1 (7.1)  4 (28.6)  5 (35.7)  2 (14.3)  2 (14.3) | ns |
| **Prior treatment failures,** mean±SD  *1-2*  *3-4*  *>4* | 4.0±2.2  1 (25.0)  2 (50.0)  1 (25.0) | 3.9±1.4  2 (15.4)  8 (61.5)  3 (23.1) | ns | 5.4±3.3  1 (6.6)  7 (46.7)  7 (46.7) | 4.2±1.7  0  13 (72.2)  5 (27.8) | ns |
| **BoNT/A responders,** n (%) | 2 (100) | 4 (100) | 0.06 | 2 (33.3) | 3 (50.0) | ns |
| **Pts with ≥1 comorbidity,** pts n (%) | 3 (21.4) | 11 (78.6) | ns | 8 (40.0) | 12 (60.0) | ns |
| **Pts with psychiatric comorbidities,** n (%) | 0 (0.0) | 3(25.0) | ns | 3 (20.0) | 4 (19.0) | ns |
| **HIT-6 score at baseline,** mean±SD | 68.7±2.7 | 68.1±3.7 | ns | 69.2±4.5 | 50.7±32.9 | **0.038** |
| **MIDAS score at baseline,** mean±SD | 83.2±33.2 | 77.5±55.8 | ns | 96.5±44.6 | 92.9±51.5 | ns |
| **Fremanezumab dosing regimen,** n (%)  *Monthly*  *Quarterly* | 1 (25.0)  3 (75.0) | 12 (92.3)  1 (7.7) | **0.022** | 10 (66.7)  5 (33.3) | 21 (100)  0 | **0.008** |

CM = chronic migraine; BMI: body mass index; MMDs: monthly migraine days; MHDs: monthly headache days; NRS= numerical rating scale; UAs = unilateral cranial autonomic symptoms; MAI = monthly analgesic intake; MO= medication overuse; BoNT/A=onabotulinum toxin A; HIT-6: Headache Impact Test-6; MIDAS=migraine disability assessment test.
